# Sedimentary Dosimetry for the Saradj-Chuko Grotto: A Cave in a Lava Tube in the North-Central Caucasus, Russia

**DOI:** 10.3390/mps3010020

**Published:** 2020-02-26

**Authors:** Bonnie A. B. Blackwell, Mehak F. Kazi, Clara L. C. Huang, Ekaterina V. Doronicheva, Liubov V. Golovanova, Vladimir B. Doronichev, Impreet K. C. Singh, Joel I. B. Blickstein

**Affiliations:** 1 Department of Chemistry, Williams College, Williamstown, MA 01267-2692, USA; 2 RFK Science Research Institute, Glenwood Landing, NY 11547-0866, USA; mackazi101@gmail.com (M.F.K.); claralhuang@gmail.com (C.L.C.H.); impreet98@gmail.com (I.K.C.S.); joelblickstein@gmail.com (J.I.B.B.); 3 ANO Laboratory of Prehistory, 199034 St. Petersburg, Russia; edoronicheva87@yandex.ru (E.V.D.); mezmay57@mail.ru (L.V.G.); labprehistory@yandex.ru (V.B.D.)

**Keywords:** ESR dating, sedimentary dosimetry, Saradj-Chuko Grotto (SCG), Russia, lava tube, Middle Paleolithic

## Abstract

Karst caves host most European Paleolithic sites. Near the Eurasian-Arabian Plate convergence in the Caucasus’ Lower Chegem Formation, Saradj-Chuko Grotto (SCG), a lava tube, contains 16 geoarchaeologically distinct horizons yielding modern to laminar obsidian-rich Middle Paleolithic (MP) assemblages. Since electron spin resonance (ESR) can date MP teeth with 2–5% uncertainty, 40 sediment samples were analyzed by neutron activation analysis to measure volumetrically averaged sedimentary dose rates. SCG’s rhyolitic ignimbrite walls produce very acidic clay-rich conglomeratic silts that retain 16–24 wt% water today. In Layers 6A-6B, the most prolific MP layers, strongly decalcified bones hinder species identification, but large ungulates inhabited deciduous interglacial forests. Unlike in karst caves, most SCG’s layers had sedimentary U concentrations >4 ppm and Th, >12 ppm, but Layer 6B2 exceeded 20.8 ppm U, and Layer 7, >5 ppm Th. Such high concentrations emit dose rates averaging ~1.9–3.7 mGy/y, but locally up to 4.1–5.0 mGy/y. Within Layer 6, dose rate variations reflect bone occurrence, necessitating that several samples must be geochemically analyzed around each tooth to ensure age accuracy. Coupled with dentinal dose rates up to 3.7–4.5 mGy/y, SCG’s maximum datable ages likely averages ~500–800 ka.

## 1. Introduction

ESR (electron spin resonance) can establish absolute (chronometric) dates for fossils, like teeth and molluscs, which range from <10 ka to >4 Ma, depending on the radiation dose rates that the fossils experience in their depositional history. Like the other trapped charge dating methods, like thermoluminescence (TL), optically stimulated luminescence (OSL), and radioluminescence (RL), ESR measures the intensity of a radiation-induced signal recorded in the datable mineral compared with the rate at which that radiation irradiates that mineral when deposited in sediment’s can date the hydroxyapatite in tooth enamel, carbonate minerals in molluscs and other invertebrates, and quartz in fault gouge and quartz-rich sediment. Used mainly in Quaternary geology and archaeology, ESR can date teeth and molluscs from some late Pliocene sites. Using teeth, ESR has been used to date many archaeological, human paleontological sites, and mammalian paleontological sites (see details in [1,2,3,4,5,6]).

In the hydroxyapatite in vertebrate tooth enamel, ESR dating uses the HAP signal at *g* = 2.0018 ± 0.0002, which has a mean signal lifetime, $\tau_{\mathrm{HAP}}^{}$ ≈ 10^19^ y ([3]; for all variables and their definitions, see Table A1). If a tooth experiences a low total radiation dose rate, *D*_Σ_(*t*), the minimum and maximum datable ESR age limits will be relative old, whereas a high *D*_Σ_(*t*) translates into much younger minimum and maximum age limits. By comparing the radiation doses accumulated in tooth enamel, 𝒜_Σ_, with *D*_Σ_(*t*) affecting the enamel, ESR dating can calculate an ESR age:

(1) $$A_{\Sigma }^{}=\int_{t_{0}^{}}^{t_{1}^{}}D_{\Sigma }^{}(t)dt$$

(2) $$A_{\Sigma }^{}=\int_{t_{0}}^{t_{1}}(D_{\mathrm{int}}(t)+D_{\mathrm{sed}}(t)+D_{\cos}(t))dt$$

where

𝒜_Σ_ = the total accumulated dose in the sample,
*D*_Σ_(*t*) = the total dose rate,
*D*_int_(*t*) = the internal dose rate from within the tooth: U, its daughters, and other radioisotopes,
*D*_sed_(*t*) = the external dose rate from sedimentary U, Th, K, and other radioisotopes,
*D*_cos_(*t*) = the external dose rate due to cosmic radiation,
*t*_1_ = the sample’s age,
*t*_0_ = today [7].

Ideally, 2–8 subsamples of enamel from each tooth are analyzed.

For subsample, the accumulated dose, 𝒜_Σ_, is determined with the additive dose method, in which 12–15 aliquots of pristine, powdered, well homogenized enamel receives added radiation doses with the highest added dose ~10 times higher than the accumulated dose measured in the 1–2 natural aliquot that receive no added irradiation. After measuring the ESR peak heights for both the naturally and artificially irradiated aliquots, the peak heights are plotted vs. their added doses to yield a growth curve whose *x*-intercept gives 𝒜_Σ_ (for example, see [6]).

In a tooth, the internal dose rate, *D*_int_(*t*), depends on the U concentration in the dental tissues, and the U uptake rate at which U was absorbed into the enamel, dentine, dental cementum, and any immediately adjacent bone. In most environments, however, neither the external radiation dose rates, *D*_sed_(*t*) nor *D*_cos_(*t*), stay constant with time. For example, *D*_cos_(*t*) drops as sediment or water accumulates above the tooth, but *D*_cos_(*t*) rises again, if erosion exposes buried teeth. Meanwhile, *D*_sed_(*t*) varies as deposition, erosion, cementation, or sedimentary diagenesis alter the sedimentary composition, its water concentration, and its thickness around a tooth. For most Middle Paleolithic teeth found in caves, however, low enamel and dentinal U concentrations often make *D*_int_(*t*) low, while *D*_cos_(*t*) tends to be small compared to *D*_sed_(*t*). Together, these factors result in dating limits that range from ~10–20 ka to ~3–4 Ma and ESR ages whose accuracy and precision depend critically depend upon the accuracy and precision in the *D*_sed_(*t*) measurements (see references in [1,2,3,4,5,6,8]).

**Figure 1:**
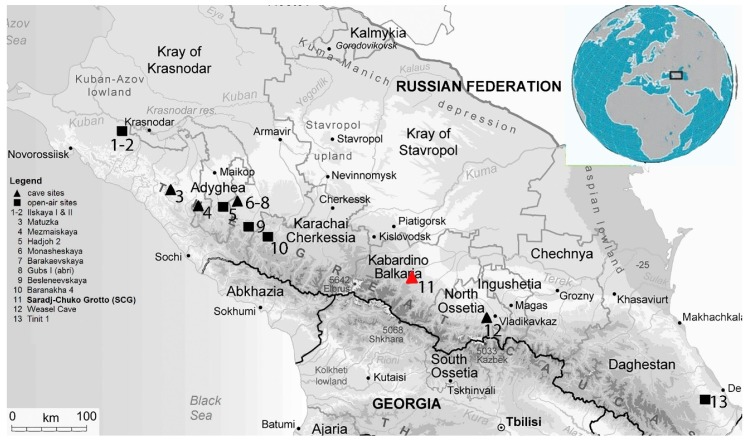
**The important stratified Middle Paleolithic sites in the Northern Caucasus, Russia.** Nestled in the Saradj-Chuko Valley, the Saradj-Chuko Grotto sits close to the fluvial divide between rivers flowing northwest into the Black Sea and those flowing southeast to the Caspian Sea. Within the Kabardino-Balkaria Republic, in the central northern Caucasus Mt., Russia, this lava tube formed in the rhyolitic ignimbrites of the Gelasian-Pliocene Lower Chegem Fm. In the later Quaternary, the grotto hosted Mousterian, other Paleolithic, and Medieval peoples.

In other depositional settings, however, the ESR age’s dating limits, accuracy, and precision can be dramatically different. This study examines how the sedimentary radioactivity in a Paleolithic site found in a lava tube affects the ESR dating analyses by examining the dosimetry on Saradj-Chuko Grotto (SCG), a newly discovered Middle Paleolithic (MP) site occurring in a lava tube in the north-central Caucasus in Kabardino-Balkaria (Figure 1; Table 1).

## 2. ESR Dating in Archaeological and Human Paleontological Sites

ESR dating is often used to date the fossils in caves *sensu lato*, especially those containing Neanderthals, other early hominins, their tool assemblages, and/or vertebrate fossils. Although open-air sites do exist that have yielded Pleistocene hominin and other vertebrate fossils, the higher propensity for open-air sites to experience erosion before, during, and after deposition means that such sites tend to lack thick stratigraphical sequences, when compared to those in caves. Lava tubes, especially the smaller tubes, tend to collapse as they undergo diagenesis (for example, see [9]), likely making it harder to find them. For Neanderthals and their tool assemblages, the majority of the multi-layer stratified sites occur in caves or abris (rock shelters). Saradj-Chuko Grotto is one of the few lava tube caves in Europe that has yielded Mousterian assemblages, whereas hundreds of karst caves in Europe, western Asia, and the Mediterranean coast of northern Africa have yielded Neanderthals, or earlier hominins, and their characteristic assemblages. In Iceland, Hawaii, and other volcanic islands, several lava tube caves have yielded recent archaeological finds (for example, see [10]). In Africa, several caves in granite, ash beds, or lava tubes have yielded hominin fossils or tool assemblages, especially those near the East African Rift. These caves include Kitum [11], Leviathan [12], the Mau Mau Caves, and Makubike [13]. Again, however, many more important multi-layered sites yielding hominins, their artefacts, or Quaternary or Pliocene vertebrates have occurred in karst caves within Africa. Many important archaological and vertebrate sites known in Eastern Asia or India also occur in karst caves, although caves in lava tubes or igneous units are also known (for example, see [14]). Few studies have examined the sedimentary dosimetry in lava tubes, unlike that in karst caves.

### Determining the External Dose Rates, D_*ext*_(t)

**Figure 2:**
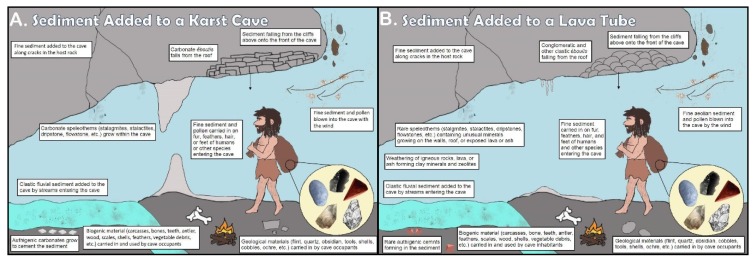
**Sediment sources for caverns.** Groundwater or surface water as fluvial or seeping in along cracks can transport sediment into caverns. Winds may add aeolian clastic or volcaniclastic sediment. Humans may contribute manuported minerals or fossils used as tools and ritual objects, but their fires also can add ash, burnt or charred wood and bone. Animals, including humans, accumulate their excretia, food debris, and their degradation products. As well as the dust and silt trapped on their feet, and in their pelts, feathers, or clothing, animals contribute their hunted or foraged prey carcasses. Plants add pollen. From the cave walls, roof, and the cliff face above the mouth come physical, chemical and biological weathering that contribute mass wasting products, including *éboulis*, as the cave stopes upward. Diagenetic processes and cementation can also add new authigenic minerals: (**A**) In karst systems, carbonate deposits, like stalagmites, stalactites, flowstone, and tufa, as well as authigenic carbonate cements usually form a significant sedimentary component. (**B**) In a grotto within a lava tube, carbonate sediment and cements will likely be insignificant compared to the clay generated by degradation of the igneous minerals or volcanic glasses.

Since Rink [1], Skinner [2], Blackwell [5], and Blackwell et al. [6] among other authors, have discussed the theory and application of ESR dating in detail, we will only discus the theory underlying the sedimentary dose rates herein. Radiation, *α*, *β*, and *γ*, from the sediment surrounding the dating sample generates *D*_sed_(*t*). In limestone caves, sediment may derive from aeolian and fluvial processes, mass wasting, seepage through cracks, stoping of the roof, non-human and human biogenic sources (Figure 2a). In a lava tube, like SCG, the sediment will lack or contain few, mostly carbonate deposits, while the more acidic sediment may destroy bone and carbonate fossils (Figure 2b). Thus, geological and archaeological sites have inhomogeneous (“lumpy”) sediment sitting within sequences of several very thin horizons with mineralogically and biologically distinct geochemistries. This generates inhomogeneous radiation dose fields that must be measured precisely and accurately to generate reliable ESR dates.

To determine a reliable *D*_sed_(*t*) experienced by a tooth, the individual dose rate, *D*_sed,*i*,*j*_(*t*), for each mineral *j*, in each layer or horizon *i* within the sphere of influence that generates the total dose rate reaching the tooth must be measured. For the *β* radiation component in sediment, that sphere of influence has a radius of ~3 mm in sediment, but the *γ* radiation sphere’s radius averages 30 cm in sediment. The radius for the sphere of influence represents the distance over which the radiation attenuates in sediment or its intensity drops below the level at which it produces a measurable effect in the sample. Radiation sources closer to the sample will produce a stronger effect on the sample than sources further away: This effect is modelled as a function of the distance squared between the source and the sample. Therefore, to assess the sedimentary *β* radiation dose rate, *D*_sed,*β*_(*t*), the fine sediment immediately attached to, or surrounding, and any other larger clasts, like *éboulis*, bone, or flint, within 3 mm of the dating sample is usually analyzed by neutron activation analysis (NAA) or other radiogeochemical analyses (i.e., fission track, X-ray fluorescence, TL dosimetry, *β* or *γ* spectrometry), while for the sedimentary *γ* radiation dose rate, *D*_sed,*γ*_(*t*), *D*_sed,*γ*,*i*_(*t*) measurements for all the layers or horizons within 30 cm of the dating sample must be calculated. Thus, since most archaeological and paleontological sites have inhomogeneous (“lumpy”) sediment, finding the most representative value for *D*_sed_(*t*) requires collecting multiple samples immediately around the sample for sedimentary dosimetry up to 30 cm away from the tooth [5]. Brennan et al. [15] discussed the issues in lumpy sites. In most sites, using γ spectrometry and TL dosimetry usually requires that the dosimetry be completed before excavation removes any sediment. Because that sedimentary inhomogeneity produces significant variation in *D*_sed_(*t*) laterally and vertically for any tooth to be dated, γ spectrometry and TL dosimetry measurements often lack the precision and accuracy needed to get the highest reliability, if not supplemented by geochemical analyses.

Since water absorbs radiation, it reduces *D*_sed_(*t*), making it essential to correct *D*_sed,*γ*,*i*_(*t*) measurements, regardless of how they were measured, if the water content varies by >5 wt% [5]. In newly excavated sites, the modern water concentration, [*W*_sed_(0)], should be measured for each layer or horizon. Nonetheless, [*W*_sed_(0)] measurements do not guarantee that [*W*_sed_(*t*)] has been constant over time, because paleoclimatic and hydrological changes may have altered the time-averaged sedimentary water concentrations $\left[{\bar{W}}_{\mathrm{sed}}^{}\left(t\right)\right]$ in the past. Therefore, geomorphological, mineralogical and geochemical data should be used to model in order to find better ${\bar{D}}_{\mathrm{sed}}^{}\left(t\right)$ and ${\bar{D}}_{\cos}^{}\left(t\right).$

To find the tooth’s time and volumetrically averaged dose rate, ${\bar{D}}_{\mathrm{sed},\gamma }^{}\left(t\right)$ derived from the γ radiation sources can then be calculated by integrating all *D*_sed,*γ*,*i*_(*t*) over the 30 cm sphere and over time [6,8,15,16]. is This includes correcting *D*_sed,*γ*,*i*_(*t*) for any absorption or leaching of radioactive elements from the various sedimentary components [16]. Analagously, ${\bar{D}}_{\mathrm{sed},\beta }^{}\left(t\right)$ is integrated over the 3 mm sphere and over time.

## 3. Saradj-Chuko Grotto (SCG), South Russia

Lying at 934 m amsl in the central Caucasus Mt., south Russia, Saradj-Chuko Grotto sits ~35 m above the Saradj-Chuko River, which is a tributary of the Kishpek River that is, in turn, a tributary of the Baksan River within the Terek River Basin in Kabardino-Balkaria. Some 70–80 km NE of Mount Elbrus, Europe’s tallest mountain, the grotto also sits 4 km south of Zayukovo and 20 km SE of Nalchik, the capital of the Kabardino-Balkaria Republic (Figure 1; [17,18,19]).

**Figure 3:**
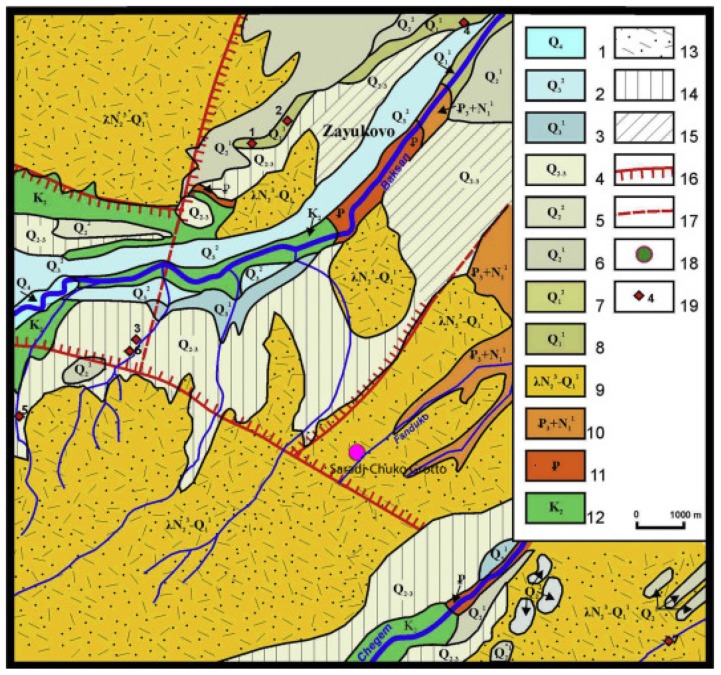
**The Geology near Saradj-Chuko Grotto.** Saradj-Chuko Grotto lies within the Lower Chegem Formation, which erupted in the extension zone between the Laba-Malka monoclinal uplift and the Tarak-Caspian Trough, during the collision zone between southern Euroasia and the Arabian Plate during the latest Pliocene and earliest Pleistocene (Gelasian). These volcaniclastic deposits extensively occur in the area. Zayukovo (Baksan) obsidian (Obsidian sites 1 and 2) was found in Layer 6B. Legend: 1–8: Quaternary units; 9: Early Gelasian-Late Pliocene Lower Chegem Fm.; 10: Middle-Upper Oligocene to Lower-Middle Miocene, Maikop Fm.; 11: Paleogene strata; 12: Upper Cretaceous strata; 13: Acidic volcanogenic deposits, mainly tuffs; 14: Undissected landslide, scree, deltaic, and fluvial units; 15: Fluvial deposits; 16: Emergent discharge, 17: Buried discharge; 18: Saradj-Chuko Grotto; 19: Obsidian and flint outcrops: 1–4: All Zayukovo (Baksan) obsidian; 5: Shtauchukua-1 flint; 6: HanaHaku-1 flint; 7: Kamenka-1 flint (adapted from Kizevalter and Karpinsky [20]).

**Figure 4:**
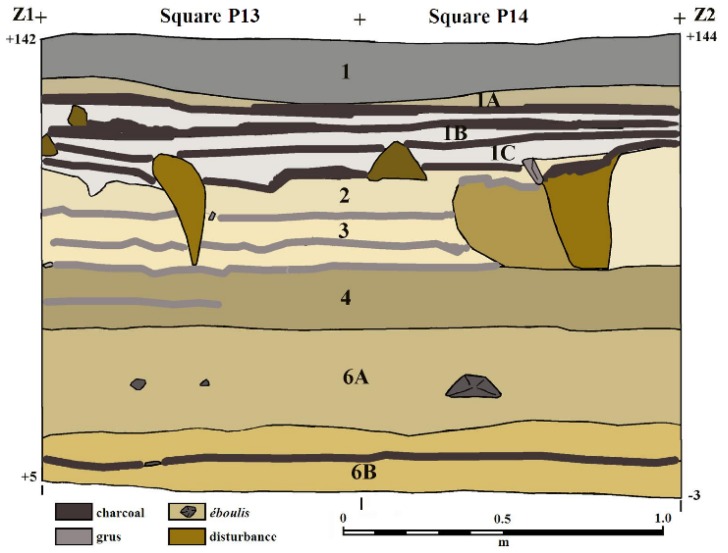
**Profile Z1–Z2, Saradj-Chuko Grotto, Russia**. In Squares Q13–Q14, nine geoarchaeological layers were identified. Cutting through Layers 2–3 in Square P17, the large disruption that begins just beneath the label for Layer 1C is likely a Medieval smelting pit or a slag discard pit.

**Figure 5:**
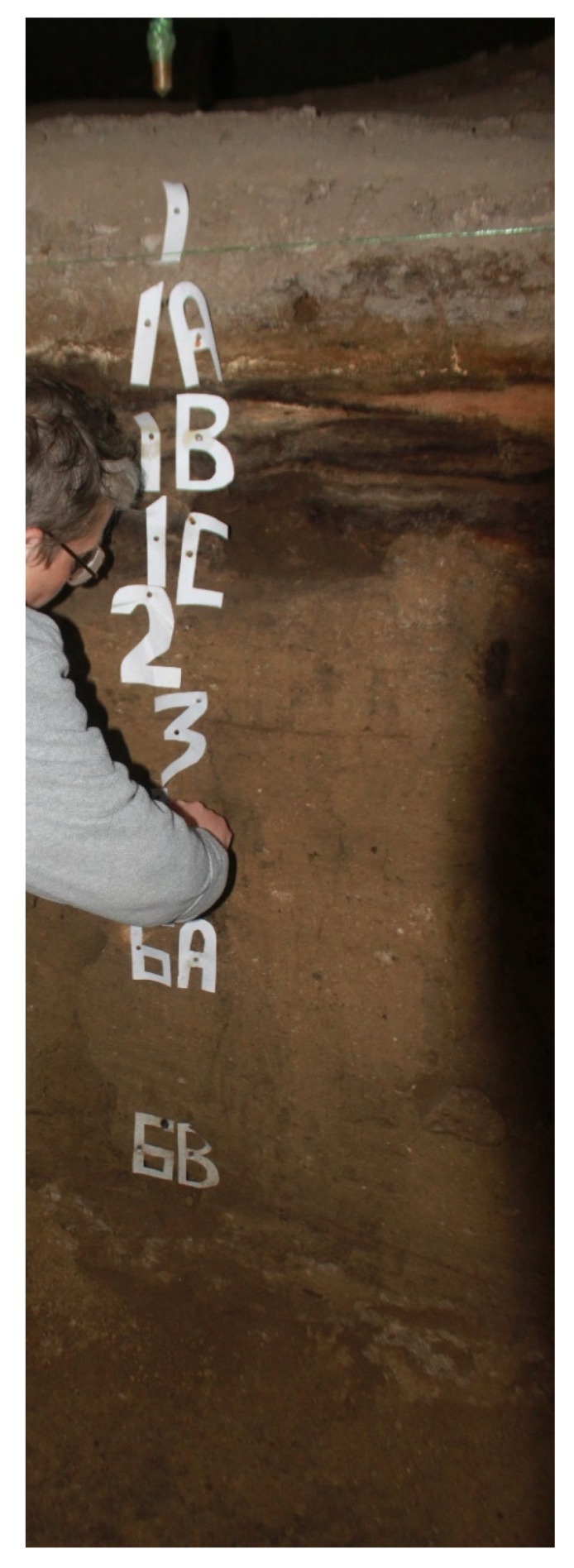
**Sampling sediment, Saradj-Chuko Grotto, Russia.** To find the sedimentary dose rates for ESR dating, 40 sediment samples were analyzed by NAA from 16 geoarchaeological horizons.

The cave formed in rhyolitic ignimbrites and tuff deposited in the Lower Chegem Formation, which erupted in the extension zone between the Laba-Malka monoclinal uplift and the Terek-Caspian Trough, in the continental convergence zone between the Saudi Arabian and Eurasian Plates (Figure 3). These Lower Chegem volcaniclastic deposits mainly comprise rhyolitic and liparitic tuffs, andesites, basalts, and rhyolitic ignimbrites ranging from a few tens to almost 500 m thick in the junction zone, although some extrusive domes, like Mt. Elbrus and Kazbek, also exist (Figure 3; [17,18,19]). Discovered in 2016, Saradj-Chuko Grotto contains 11 distinct geoarchaeological horizons ranging from modern, Medieval, Roman to Middle Paleolithic deposits (Figure 4 and Figure 5). Averaging 5–6 cm thick, Layer 1 contains a grey sandy silt that dips to the NE. With few animal fossils and slag, mixed Medieval ceramic and several obsidian pieces suggest a bioturbated deposit. Layers 1A–C comprise yellow, sandy silt units mixed with charcoal and ash [17,18,19].

Dipping to the west, Layer 2 ranges from 11 to 24 cm of yellow, sandy silt (Figure 4 and Figure 5). Although Layer 2 yielded a few mammal bones, it lacks artefacts. Also dipping to the west, Layer 3 averages 12–17 cm thick, with yellow sandy silt interspersed with three gruss horizons. Layer 3 yielded a few mammal fossils and obsidian artefacts, one of which was a Middle Paleolithic bifacial tool. Comprising 14–30 cm of grey-brown silt dipping to the west, Layer 4 also yielded obsidian artefacts and several mammal bones. Actually a tectonic fissure, Layer 5 produced flakes, but few fauna in a dark brown-black sandy silt [17,18,19].

Averaging 30–40 cm thick dipping to the west, Layer 6A comprises gray sandy silt with some tuff *éboulis* likely fallen from the roof (Figure 4 and Figure 5). During the 2017 excavations, Layer 6A yielded 72 artefacts. Of these, most were made on obsidian, with only two on flint, and one on silicified limestone. With no formal tools or cores, eight flakes, 46 shatters, and 18 chips were identified, probably attributable to the Middle Paleolithic. Most of the obsidian tools and chips were made on Zayukovo (Baksan) obsidian [17,18,19].

At 20 cm thick, Layer 6B contains dark brown silt with ignimbrite pebbles and tuff (Figure 4 and Figure 5). Some organic matter contained secondary iron hydroxides, while some bones had decomposed to decalcified phosphate clusters. In 2017, Layer 6B yielded 1591 lithic artefacts, 98% of which were made on obsidian, again mainly Zayukovo obsidian [17,18,19].

Undoubtedly, hominins knapped local obsidian in SCG, but any flint artefacts had been imported ready to use. High quality grey, black, and pink flint was also available along the Baksan, Chegem, and Kamenka River valleys (Figure 3). The flint may have come from local sources, such as HanaHaku and Shtaucukua 5–7 km to the northwest, and from Kamenka to the southeast. Inhabitants rejuvenated flint artefacts at the site. In Layer 6B, the few retouched tools included simple side- scrapers, rare diagonal and transverse sidescrapers, convergent tools, and Mousterian points. Neanderthals likely made this Levallois-laminar Mousterian industry [17,18,19].

Where visible, Layer 7 ranges >20–30 cm thick (Figure 5), but it has not been fully excavated, iron hydroxide has cemented the archaeologically sterile ignimbrite. Excavations have yet not hit the grotto floor [17,18,19].

In Layers 6B and 7, pollen analyses found many tree and shrub grains, including *Alnus glutinosa*, *Ulmus campestris*, and *Castanea sativa*, with rare Compositae, Poaceaea, and Apiacae. These layers were deposited during an interglacial period, most likely Marine Isotope Stage (MIS) 5 when hazel-hornbeam-oak forests with oriental beech, chestnuts, and walnuts surrounded the cave. *Sula* and *Strobus* suggest that the minimum winter temperature locally reached −9 °C, while precipitation averaged 80–150 cm/y. These mixed deciduous and coniferous forests provided hominins with complex dietary choices [17,18,19].

Within Layer 6B, the very fragile bones ranged from whitish grey to yellowish-orangish red. In some, the original bone shape (“ghosts”) could be discerned when first found, but they had been decalcified enough that they disintegrated into tiny flakes, spots, or dust if disturbed. Most intact bones had been broken or cracked before deposition. Thus, taxonomically or anatomically (typing) identifying many bones was impossible. Some bones, however, had been heated, charred, or calcined, hinting the animals had likely been hunted by Neanderthals [17,18,19].

## 4. Method

Since this study does not focus on the ESR dating age analyses, only a brief description of the tooth’s preparation for ESR dating will suffice here. All samples were prepared using standard protocols for a Class 10,000 clean lab. To reduce cross-sample contamination, all glass- and plasticware were soaked in 6 M HCl(*aq*) and rinsed ≥15 times with doubly distilled, deionized water to remove all Cl^−^ ions [21].

After measuring their thicknesses with a CD-4C digital caliper, the tooth was split into four subsamples with a diamond-tipped Dremel drill, and each subsample cleaned of any attached sediment or dentine, which were removed and saved for NAA. After measuring enamel thicknesses in 30–50 places with a Mitutoyo IP-C112E micrometer, 20 µm were shaved off both enamel sides to remove the externally *α*-dosed enamel and thicknesses were remeasured. After powdering the enamel to 200–400 mesh (38–76 μm) in an agate mortar and pestle, it was split into 13–16 identical aliquots, each weighing 20.0 ± 0.1 mg [21].

Immediately after collection in the field (Figure 5), sediment samples were doubly sealed in ziplock bags. Samples with sharp gravel were triply bagged. Before powdering the sediment for NAA, ~30 g of sediment was sampled for [*W*_sed_(0)] immediately after opening the bags. To calculate [*W*_sed_(0)], the sediment mass was measured and gently heated at ~50 °C for several days until no mass changes were recorded to find the mass loss.

To measure [*U*_sed_], [*Th*_sed_], and [*K*_sed_], all associated sediment were powdered to ≤500 mesh and analyzed by neutron activation analysis (NAA). In JT5, 1–2 dentines/subsample and enamels/subsample were analyzed for their U concentrations, [*U*_den_] and [*U*_en_] respectively. After a 60.0 s irradiation and a 10.0 s delay, U was counted for 60.0 s in a delayed neutron counting system. Th and K were counted for 20.0 min in a γ counter. Th was counted after a 1.0 h irradiated and a 7.0 day delay. K had a 24–30 h delay after a 60.0 s irradiation. To ensure accuracy, all results were calibrated against NIST Standard 1633B [21].

Each *D*_sed,*i*,*j*_(*t*) and its error for each mineral, *j*, in Layer *i*, was calculated from [*U*_sed_], [*Th*_sed_], [*K*_sed_], and [*W*_sed_(0)] [22], assuming no cosmic dose rate contribution, using both Rosy v. 1.4.2 [23] and Data [24]. Both these programs calculate *D*_sed,*i*,*j*_(*t*) as seen by the sample sitting in a sphere of homogeneous sediment corrected for radiation backscattering and for *β* and *γ*, but not *α*, attenuation, due to the sedimentary and tissue water concentrations, tissue density, and thicknesses from sediment and tissues. ${\bar{D}}_{\mathrm{sed}}^{}\left(t\right)$ were calculated by using the program VolSed v. 2.2 that integrates all the applicable *D*_sed,*i*,*j*_(*t*) values within 3 mm for the *β* dosimetry or 30 cm for the γ dosimetry respectively over their applicable sedimentary units, weighted by their volume and distance from the sample. Distances between sedimentary components and a tooth was determined visually in the field, from the total station data, and from the site photographs. For the saturation simulations and modelling, ${\bar{D}}_{\cos}^{}\left(t\right)$ were calculated assuming ramped box models [7] using Rosy v. 1.4.2, and integrated over time since the simulated deposition using the program AgeTimeAv v. 4.4. Comparisons between the other dosimetry methodologies, namely “instantaneous” γ and TL dosimetry, with the geochemical analyses described here, show that insignificant differences occurred between the three dosimetric methods (for example, see [5,6,25], and more references therein).

## 5. Results and Discussion

**Figure 6:**
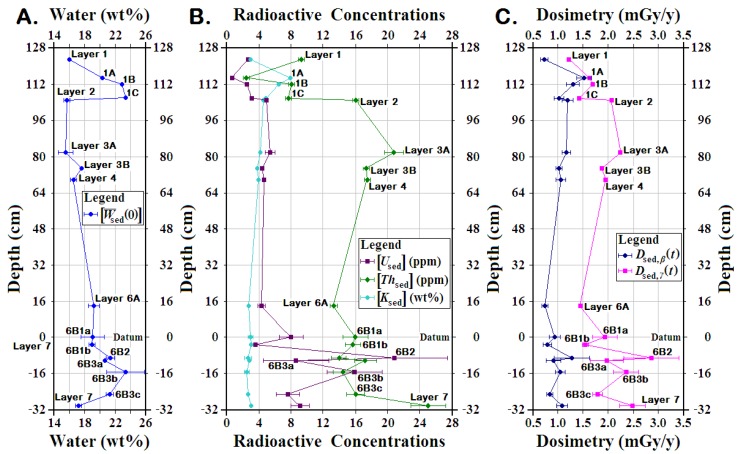
**Factors affecting sedimentary dosimetry vs. depth, Saradj-Chuko Grotto (SCG), Russia.** Mean factors plotted here show that significant changes occur with depth in the sediment at SCG: (**A**) Mean modern sedimentary water concentration, $\left[{\bar{W}}_{\mathrm{sed}}^{}\left(0\right)\right]$, vs. depth: Layers 1B, 1C, and 6B3b had highest mean sedimentary water concentrations, all of which exceeded 22 wt%, while Layers 3A and 2 had the lowest averaging <16 wt%. Layers 1, 4, and 7 also had water concentrations at 16–17 wt%. Within Layer 6, water concentrations ranged from 18.9 ± 0.3 to 23.8 ± 2.4 wt%, with higher uncertainties than seen in other layers. (**B**) Radioactive elemental concentrations vs. depth: For all but Layers 1A, 1B, 1C, and 2, mean [K_sed_] stayed below 4 wt%, with a low at 2.5 ± 0.2 wt%. Layer 1A had the highest [*K*_sed_] occurred at 7.9 ± 0.2 wt%, but the lowest [*Th*_sed_] and [*U*_sed_] at 2.28 ± 0.09 and 0.60 ± 0.02 ppm respectively. Below Layers 1–1C, all the mean [*Th*_sed_] exceeded 12 ppm, while the mean [*U*_sed_] exceeded 3.5 ppm. Within Layer 6, significant variations in both [*U*_sed_] and [*Th*_sed_] and their uncertainties occurred. (**C**) Sedimentary dosimetry vs. depth: While both the highest *D*_sed,*β*_(*t*) and *D*_sed,*γ*_(*t*) within Layer 6 occurred in 6B2, Layers 6B1b and 6A both had very low *D*_sed,*β*_(*t*) and *D*_sed,*γ*_(*t*). Since both *D*_sed,*β*_(*t*) and *D*_sed,*γ*_(*t*) showed substantial variation and high uncertainties from one horizon to the next, the means, ${\bar{D}}_{\mathrm{sed},\beta ,i,j}^{\mathrm{BG}}\left(t\right)$ and ${\bar{D}}_{\mathrm{sed},\gamma ,i,j}^{\mathrm{BG}}\left(t\right),$ could not be used to calculate the specific volumetrically averaged dose rates, ${\bar{D}}_{\mathrm{sed},\beta ,i}^{}\left(t\right)$ or ${\bar{D}}_{\mathrm{sed},\gamma ,i}^{}\left(t\right),$ near each tooth. Instead, several samples with 30 cm of each tooth must be measured, which will increase the cost to date each tooth.

In Saradj-Chuko Grotto, water, U, Th, and K concentrations were measured for 16 different layers or horizons, from which *D*_sed,*β*_(*t*) and *D*_sed,*γ*_(*t*) were calculated (Table 2). To see the trends, means for the four concentrations and the two dose rates were plotted vs. depth (Figure 6).

### 5.1. Sedimentary Geochemistry

Throughout the layers at SCG, the sediment retained high modern water concentrations, [*W*_sed_(0)] (Table 2; Figure 6a). Layers 3A and 2 had the lowest mean [*W*_sed_(0)] at 15.5 ± 0.9 and 15.6 ± 0.4 wt% respectively, while Layer 1 averaged 15.9 ± 0.2 wt%, and Layer 4, at 16.5 ± 0.3 wt%. Probably due to its higher sand concentrations and less clay, Layer 7 had a water concentration at 17.1 ± 0.3 wt%. Within Layer 6, water concentrations ranged from 18.9 ± 0.3 to 23.8 ± 2.4 wt%, with higher uncertainties than seen in other layers. Meanwhile, Layers 1B, 1C, and 6B3b all had >22 wt% [*W*_sed_(0)]. Within Layer 6, [*W*_sed_(0)] ranged from 18.9 ± 0.3 to 23.8 ± 2.4 wt%, with higher uncertainties than seen in other layers. In most karst cave sediment, [*W*_sed_(0)] tends to average 5–15 wt% [5,6,8,25,26,27,28,29,30,31,32,33]. At [*W*_sed_(0)] >12–15 wt%, the sediment feels damp, which tends to discourage both long-term human inhabitation and cave bear hibernation. Undoubtedly, the high clay concentrations in most of SCG’s sedimentary layers ensures that they stayed wet, which also promoted higher degradation of bone and other the organic remains, as well as the igneous minerals found in the ignimbrite constituting the cave rocks. Whether under different climate regimes, the sediment may not have stayed as wet as it is now. Nonetheless, wet sediment might have discouraged hominin and cave bear inhabitation in the grotto during all but the driest seasons and the coldest glacial periods. With the high bone dissolution, the dental seasonal analyses needed to answer this question have yet to be completed.

The SCG sediment also contained high K concentrations, [*K*_sed_] (Table 2; Figure 6b). In both Layers 1B and 1C, [*K*_sed_] exceeded 6.5 wt%, but in Layer 6B3c, [*K*_sed_] averaged a low of 2.47 ± 0.24 ppm. Nonetheless, throughout SCG, [*K*_sed_] at 2.5–4.0 wt% more than doubles the typical [*K*_sed_] at 0.5–2.0 wt% seen in most karst cave sediment [5,6,8,25,26,27,28,29,30,31,32,33]. Thus, the high [*K*_sed_] also leads to higher *D*_sed*,*_*_β_*(*t*) (see below).

Except for Layer 6B1b, all the layers deeper than Layer 1C had sedimentary U concentrations, [*U*_sed_], >4 ppm (Table 2; Figure 6b). Layer 1A had the lowest U concentrations at 0.60 ± 0.02 ppm, but Layer 6B2′s [*U*_sed_] exceeded 20.8 ppm U, with 2018SCG61 at 29.73 ± 0.02 ppm (Table 2l). In SCG, its [*U*_sed_] values average 4–6 ppm for those layers above Layer 6, but below, [*U*_sed_] generally exceeded 7.5 ppm, except for Layer 6B1b. In karst caves, the sediment tends to have [*U*_sed_] ≲4 ppm [2,5,6,8,25,26,27,28,29,30,31,32,33] namely ≤50% of that seen in SCG.

Below Layer 1C, all the mean Th concentrations, [*Th*_sed_], exceeded 12 ppm (Table 2; Figure 6b). The lowest [*Th*_sed_] occurred in Layer 1A at 2.28 ± 0.09 ppm, while Layer 7 had highest [*Th*_sed_] at 25.05 ± 2.15 ppm. In typical karst caves, [*Th*_sed_] usually ranges from 3 to 8 ppm, but the carbonate rocks often have [*Th*_sed_] near or below NAA [*Th*_sed_] detection limits [5,6,8,25,26,27,28,29,30,31,32,33]. Hence, SCG’s [*Th*_sed_] ranged from 150% to >300% more than typically seen in karst cave sediment.

Interestingly, within Layer 6, large variations in both [*U*_sed_] and [*Th*_sed_] occurred (Table 2i–o; Figure 6b). Due to the high [*W*_sed_(0)], and its igneous source rocks that weather into sediment with high acidity, the SCG sediment contained up to 40–50% clay in many horizons. That acidity contributed to high dissolution rates for bone and other organic tissues, as shown by the prevalence of the bone ghosts seen in many layers. Once exposed to U in the sediment, however, bone, dentine, and dental cementum begin to scavenge U rapidly [34,35,36,37,38,39]. Certainly, samples with higher bone concentrations had more [*U*_sed_], coupled with somewhat lower [*Th*_sed_]. These horizons also yielded the higher artefact numbers. Likely, in these horizons, the hominins contributed more bone and dental tissues to the sediment than in other horizons. Without a good proxy for the initial bone concentrations, however, estimating how much [*U*_sed,os_] derived from U scavenging by bone and dental issues from the groundwater compared to U scavenged from [*U*_sed,igrx_], including [*U*_sed,*éb*_], after igneous rock dissolution is difficult. Nor can we estimate how high [*U*_sed_] might have been for each horizon before the bones began to dissolve and release [*U*_sed,os_] again to the sediment, from where it may have been subsequently leached.

### 5.2. Sedimentary Dose Rates

Overall, the SCG’s *D*_sed,*β*_(*t*) ranged from 0.727 ± 0.064 to 1.519 ± 0.064 mGy/y, while *D*_sed,*γ*_(*t*) varied from a low of 1.212 ± 0.016 mGy/y to a high of 2.851 ± 0.539 mGy/y (Figure 6c; Table 2). The highest *D*_sed,*β*_(*t*) and *D*_sed,*γ*_(*t*) occurred in Layers 2–4 and some horizons in Layers 6 and 7.

In Layer 1, although [*W*_sed_(0)] rose with depth, *D*_sed,*β*_(*t*) reached a local maximum in Layer 1A due to high [*K*_sed_] and *D*_sed,*γ*_(*t*) in Layer 1B due to [*Th*_sed_] (Figure 6; Table 2). In Layers 2–3, lower high [*W*_sed_(0)] coupled with high [*Th*_sed_] produced high *D*_sed,*β*_(*t*) and *D*_sed,*γ*_(*t*). In Layer 7, low [*W*_sed_(0)] mixed with both high [*U*_sed_] and [*Th*_sed_] again yielded high *D*_sed,*β*_(*t*) and *D*_sed,*γ*_(*t*).

Within Layer 6, Layers 6a, 6B1b, 6B3a, and 6B3c had lower *D*_sed,*β*_(*t*) and *D*_sed,*γ*_(*t*), while Layers 6B1a, 6B2, and 6B3b gave higher *D*_sed,*β*_(*t*) and *D*_sed,*γ*_(*t*) (Figure 6c; Table 2i–o). In Layers 6a and 6B1b, moderately low [*W*_sed_(0)] combined with low [*U*_sed_] to make low *D*_sed,*β*_(*t*) and *D*_sed,*γ*_(*t*). Despite higher [*W*_sed_(0)], the very high [*U*_sed_] in Layers 6B2 and 6B3b produced the highest and second highest *D*_sed,*β*_(*t*) and *D*_sed,*γ*_(*t*) within Layer 6.

**Figure 7:**
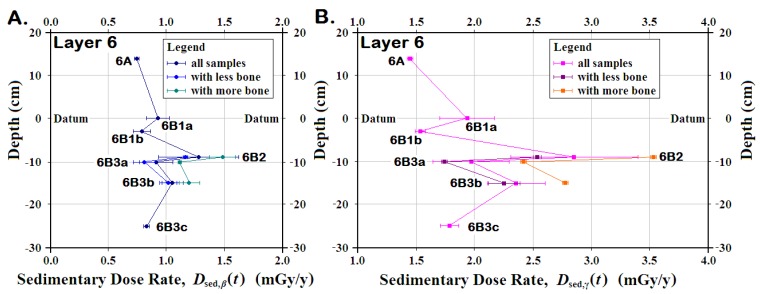
**The effect of bone in the sedimentary dose rates, *D*_sed_(*t*), at Saradj-Chuko Grotto, Russia**. Within Layer 6 at Saradj-Chuko, having more bone in the sediment produced: (**A**) higher *β* sedimentary dose rates, *D*_sed,*β*_(*t*) (**B**) higher γ sedimentary dose rates, *D*_sed,*γ*_(*t*) Bone-rich samples had up to 0.32 mGy/y for *D*_sed,*β*_(*t*) and 1.0 mGy/y for *D*_sed,*β*_(*t*). If bone-rich samples were removed from the means for *D*_sed,*β*_(*t*)and *D*_sed,*β*_(*t*), the resulting *D*_sed,*β*_(*t*)and *D*_sed,*γ*_(*t*) had lower mean rates with significantly smaller uncertainties. Thus, knowing the precise locations for bone-rich sediment will increase the precision for the dose rates.

Within Layer 6, *D*_sed,*β*_(*t*), *D*_sed,*γ*_(*t*), and their uncertainties varied greatly from horizon to horizon, due in part to [*U*_sed,os_] associated with the sedimentary bone concentrations (Figure 6c; Table 2i–o). Since [*U*_sed_] strongly affected both *D*_sed,*β*_(*t*) and *D*_sed,*γ*_(*t*), the effect of [*U*_sed,os_] was examined by plotting the means for both *D*_sed,*β*_(*t*) and *D*_sed,*γ*_(*t*) using all samples analyzed from the layer, as well as the means in the samples having higher amounts of bone, and those without the highest [*U*_sed,os_] samples (Figure 7; Table 2i–o). In each of the three horizons, removing the sample with the highest [*U*_sed_] left a smaller mean with a significantly smaller uncertainty. Nonetheless, the mean for Layer 6B2 without the highest [*U*_sed_] still had much higher *D*_sed,*β*_(*t*) and *D*_sed,*γ*_(*t*) than for all other horizons and comparable those seen in Layer 7, which lacks hominin artefacts.

In karst caves, *D*_sed,*β*_(*t*) typically ranges from 100 to 500 µGy/y, while *D*_sed,*γ*_(*t*) varies from 300 to 1000 µGy/y, partly due to the limestone and *éboulis* that tend to range at 30–60 µGy/y for *D*_sed,*éb*,*β*_(*t*) and 100–250 µGy/y for *D*_sed,*éb,* γ_(*t*) [5,6,8,16,25,26,27,28,29,30,31,32,33]. By comparison, at SCG, both *D*_sed,*β*_(*t*) and *D*_sed,*γ*_(*t*) range 3–15 times higher.

### 5.3. The Effects on the ESR Ages

With such high *D*_sed_(*t*) in several horizons and their high variations within Layer 6, the volumetrically averaged sedimentary dose rates, ${\bar{D}}_{\mathrm{sed}}^{}\left(t\right),$ will need to be calculated using the individual *D*_sed*,i,j*_(*t*) for each layer and sedimentary component within the 3 mm and 30 cm spheres of influence around each tooth. Using the means, ${\bar{D}}_{\mathrm{sed},\beta ,i,j}^{\mathrm{BG}}\left(t\right)$ and ${\bar{D}}_{\mathrm{sed},\gamma ,i,j}^{\mathrm{BG}}\left(t\right),$ for each horizon will not provide, ${\bar{D}}_{\mathrm{sed}}^{}\left(t\right)$ that will be accurate and precise enough to get the most reliable ESR ages. Thus, several individual sediment samples near each tooth must be tested by NAA. This could dramatically increase the costs for dating each tooth.

To test the dentinal dose rates, eight subsamples from JT5 were analyzed for its U concentrations, [*U*_den_] (Table 3). Not surprisingly, both [*U*_inden_] and [*U*_outden_] ranged from 136.12 to 162.38 ± 0.02 ppm, which produce *D*_den_(*t*) ranging from 3.751 ± 0.270 to 4.475 ± 0.322 mGy/y assuming an early U uptake model in the dentine. Again, [*U*_den_] tend to range 100–150 ppm higher than comparable [*U*_den_] values seen in dentine from karst caves [5,6,8,25,26,27,28,29,30,31,32,33,38,39]. JT5′s *D*_den_(*t*) emits as much as ~2.5–4.0 mGy/y, significantly more than in the comparable dentine seen in Middle Paleolithic teeth collected from karst caves.

In humans, the lethal dose for 50% of people tested, LD_50_, is ≲4 Gy of radiation, although a dose as low as 0.25 Gy produces measurable effects in the body [40]. With a combined *D*_sed_(*t*) averaging from ~1.9 to 3.7 mGy/y, but locally as high as 4.1–5.0 mGy/y, coupled with *D*_den_(*t*) as high as 3.7–4.5 mGy/y, hominins living in SCG received measurable effects after as few as ~26 years at the highest dose rates, assuming that no areas in the cave have a higher dose rate. Although they would not likely accumulate lethal doses in a lifetime, especially if the wet sediment discouraged long-term inhabitation, the effects on mutation and cancer rates likely affected people who inhabited SCG for short times periodically over many years or those visiting frequently.

Since both *D*_sed_(*t*) and *D*_den_(*t*) are so high, any tooth from SCG will have a much higher accumulated dose, 𝒜_Σ_, than a tooth of comparable age from a karst cave. Because the precision with which 𝒜_Σ_ can be calculated drops as 𝒜_Σ_ rises, the precision for the ESR ages for the SCG teeth also drops with the rising 𝒜_Σ_. For most teeth, full saturation of the HAP signal occurs at ~13–22 kGy, but must be tested in each. At the highest *D*_sed_(*t*) and *D*_den_(*t*) seen in SCG, teeth might reach their full saturation dose, 𝒜_sat_, within 250 ka of being deposited. More realistically, given that neither all the horizons within the spheres of influence around each tooth are likely to emit the highest *D*_sed_(*t*) nor are all *D*_den_(*t*) will have the highest concentrations, the maximum dating age could be <500–800 ka. If the teeth date <200–250 ka (i.e., Marine Isotope Stage, MIS, 7) or even <360–420 ka (i.e., MIS 11), reliable ages should be calculable. Given that the assemblages in the oldest layers resemble closely the Mousterian, one would expect their teeth to post-date 200 ka. If, however, the cave contains teeth deposited older than MIS 15, their calculated ages might represent minimum ages, because their teeth might have reached their 𝒜_sat_. Should more archaeological layers occur below Layer 7, the potential for encountering teeth that might have reached saturation increases.

**Figure 8:**
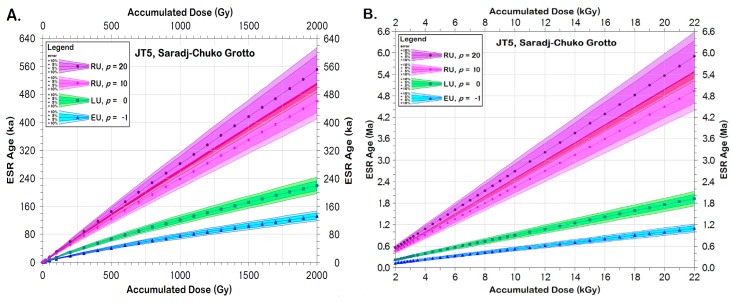
**Calculated ages vs. accumulated doses,𝒜_Σ_, JT5, Saradj-Chuko Grotto, Russia**. Using the preliminary volumetrically averaged sedimentary dose rates, ${\bar{D}}_{\mathrm{sed},\beta }^{}\left(t\right)$ and ${\bar{D}}_{\mathrm{sed},\gamma }^{}\left(t\right),$ ESR ages were calculated for JT5 at: (**A**) 𝒜_Σ_ = 0.0–2.0 kGy (**B**) 𝒜_Σ_ = 2.0–22.0 kGy As 𝒜_Σ_ rises, so do the calculated ages, with a nearly linear function. Assuming that an early U uptake model (EU, *p* = −1) describes the U uptake rate into the tooth, JT5 would have 𝒜_Σ_ = 0.99–1.96 kGy if it dates to MIS 5. Under EU, JT5 would reach its saturation dose, 𝒜_sat_, after ~665–1100 ky following its initial deposition. Using a linear U uptake model (LU, *p* = 0), JT5 would have 𝒜_Σ_ = 0.58–1.06 kGy if it dates to MIS 5. Under LU, JT5 would reach its 𝒜_sat_ at ~1.16–1.92 My after its initial deposition. Assuming a recent U uptake (RU) model with an U uptake rate, *p* = 10, an age for JT5 dating to MIS 5 would have 𝒜_Σ_ = 280–520 Gy. Assuming *p* = 10 (RU), JT5 would likely reach its 𝒜_sat_ after ~2.92–4.94 My. Assuming *p* = 20, a tooth from MIS 5 would have 𝒜_Σ_ = 250–240 Gy, and reach its 𝒜_sat_ after ~3.59–5.90 My in the sediment. Thus, this analysis shows that JT5 will give definitive ESR ages regardless of its actual U uptake model. For teeth found in other layers with higher ${\bar{D}}_{\mathrm{sed},\beta }^{}\left(t\right)$ and ${\bar{D}}_{\mathrm{sed},\gamma }^{}\left(t\right)$, or for those found in thicker bone-rich horizons, however, their 𝒜_Σ_ may approach the saturation dose, 𝒜_sat_, too closely to distinguish its 𝒜_Σ_ from 𝒜_sat_. That would make it impossible to calculate anything other than a minimum age limit.

At a depth of +11 cm, JT5 sat within Layer 6B1a, just above the boundary with Layer 6B1b. For the *β* dosimetry, both Layers 6b1a and 6B1b fell within JT5′s sphere of influence, giving its preliminary ${\bar{D}}_{\mathrm{sed},\beta }^{}\left(t\right)$ = 955 ± 91 μGy/y. For the γ dosimetry, 2 cm of Layer 4 and 18 cm of Layer 6A overlay JT5, while 18 cm of Layer 6B2 lay below Layer 6B1b. After estimating and correcting for the amount of bone and rooffall around JT5, its preliminary ${\bar{D}}_{\mathrm{sed},\gamma }^{}\left(t\right)$ = 2000 ± 109 μGy/y. Final calculations must await excavation of nearby quadrants to assess the currently hidden sediment nearby and its inhomogeneous components. If JT5 was deposited during MIS 5 (i.e., ~74–128 ka), as the palynological analyses suggest [18], JT5′s accumulated dose, 𝒜_Σ_, would be expected to lie between 580 and 1080 Grays, assuming a linear U uptake model (LU; Figure 8a), which would allow an definite age determination for JT5, rather than a minimum age estimate. Assuming LU, JT5 would likely reach 𝒜_sat_ at ages between 1.16 and 1.92 My after its initial deposition (Figure 8b). With the crystal damage rates produced by the high ionizing radiation fields bathing the teeth and the dentinal U concentrations (Table 3), however, the teeth might have uptaken U more frequently as the tooth aged. Thus, an RU model, with the uptake rate, *p* > 0, would better model the tooth’s U uptake history. If so, JT5′s MIS 5 ages would be older, and it would likely reach its 𝒜_sat_ if it had been deposited earlier in the Quaternary than would be generated by assuming LU.

## 6. Conclusions

Unlike typical karst limestone caves, Saradj-Chuko Grotto nestles in rhyolitic ignimbrite. Due to its calcium carbonate, limestone buffers the sediment and its groundwater, but the rhyolitic ignimbrite at SCG lacks an effective geochemical buffer. Thus, in situ degradation of the ignimbrite has made the sediment very acidic. Thus, the acidic sediment contains high clay concentrations from the degraded silicates and obsidian in the lava. Acting as an aquatard, the clay also retains water well, producing [*W*_sed_(0)] higher than 16 wt% on average, but as high as ~24 wt%. Since clays adsorb Th, the SCG sediment contains 10–20 ppm more [*Th*_sed_] than those seen in most karst cave sediment. At SCG, [*K*_sed_] concentrations also far exceeded those in karst caves. Dissolution of bones that scavenged [*U*_sed_] from the local groundwater has produced [*U*_sed_] up to ~30 ppm, SCG’s [*U*_sed_] averaged 5–10 times higher than [*U*_sed_] seen in most karst caves. In SCG, ${\bar{D}}_{\mathrm{sed},\beta }^{}\left(t\right)$ averaged from 0.727 ± 0.064 to 1.519 ± 0.142 mGy/y and ${\bar{D}}_{\mathrm{sed},\gamma }^{}\left(t\right)$ from 1.212 ± 0.016 to 2.987 ± 0.024 mGy/y. Namely, SCG’s ${\bar{D}}_{\mathrm{sed},\beta }^{}\left(t\right)$ and ${\bar{D}}_{\mathrm{sed},\gamma }^{}\left(t\right)$ both exceed those seen in typical karst caves by 200–300%. In the fossils, the dentine and bone scavenged U from the uraniferous groundwater bathing the sediment, leading to high *D*_den_(*t*) measures. With high [*U*_den_] near 130–160 ppm, *D*_den_(*t*) also contribute radiation at 2–4 times faster than typical seen in karst caves. Therefore, for some SCG teeth found in the horizons rich in bone where the highest ${\bar{D}}_{\mathrm{sed}}^{}\left(t\right)$ occur, a viable maximum datable age may be as small as 0.25–0.8 Ma. Hominins living in SCG might have begun to experience medical effects from the high radiation rates within a few decades. People excavating in SCG should also be monitored with personal dosimeters.

## Figures and Tables

**Table 1:** Teeth from Saradj-Chucko Grotto in the Study.

Number			Location					Sample Type	
ESR	Field	Plan	Layer (Horizon)	Square	South, X (cm)	West, Y (cm)	Depth, Z (cm)	Species	Tooth
JT5	2018SCG35	4	6B (1)	P17	34	48	11	herbivore	cheek

**Table 2:** Sedimentary Radioactivity, Saradj-Chuko Cave, Russia.

	Location						Concentrations				Sedimentary Dose Rates ^1^	
Sample	Layer	Square	N–S, X (cm)	E–W, Y (cm)	Depth, Z (cm)		Water (wt%)	U (ppm)	Th (ppm)	K (wt%)	$D_{\mathrm{sed},\beta }^{BG}{\left(t\right)}^{2}$ (mGy/y)	$D_{\mathrm{sed},\gamma }^{BG}{\left(t\right)}^{3}$ (mGy/y)
a. Layer 1												
2018SCG11	1	Q17	72	98	+123		15.9	2.62	9.25	2.86	0.727	1.212
bulk sediment						±	0.2	0.02	0.18	0.07	0.064	0.016
b. Layer 1A												
2018SCG10	1A	Q14	40	97	+115		20.3	0.60	2.28	7.90	1.519	1.627
bulk sediment						±	0.2	0.02	0.09	0.20	0.142	0.037
c. Layer 1B												
2018SCG09	1B	Q14	35	97	+112		22.9	2.45	8.09	6.44	1.290	1.688
bulk sediment						±	0.1	0.02	0.15	0.18	0.119	0.033
d. Layer 1C												
2018SCG08	1C	Q14	43	97	+106		23.3	3.04	7.61	4.88	1.019	1.429
bulk sediment						±	0.2	0.02	0.17	0.13	0.093	0.024
e. Layer 2												
2018SCG06	2	Q15	4	96	+106		15.6	4.87	16.01	4.54	1.193	2.055
bulk sediment						±	0.4	0.02	0.27	0.12	0.105	0.029
f. Layer 3A												
2018SCG07	3A	Q17	23	97	+83		14.6	4.81	19.74	4.31	1.185	2.193
bulk sediment						±	0.1	0.02	0.35	0.11	0.104	0.027
2018SCG19	3A	Q17	58	13	+78		16.4	5.93	21.93	4.00	1.148	2.277
bulk sediment						±	0.1	0.02	0.37	0.10	0.099	0.026
bulk sediment	3A	Q17			+86		15.5	5.37	20.84	4.16	1.166	2.238
mean (*n* = 2)					− +78	±	0.9	0.56	1.09	0.16	0.072	0.019
g. Layer 3B												
2018SCG17a	3B	Q17	64	17	+79		17.5	4.28	17.82	3.84	1.014	1.889
bulk sediment						±	0.1	0.02	0.31	0.10	0.089	0.023
2018SCG18	3B	Q17	54	13	+78		17.2	4.42	17.13	3.74	1.000	1.861
bulk sediment						±	0.1	0.02	0.31	0.10	0.088	0.023
2018SCG20	3B	Q17	60	17	+72		17.8	4.31	16.99	3.96	1.029	1.873
bulk sediment						±	0.2	0.02	0.31	0.10	0.090	0.024
bulk sediment	3B	Q17			+79		17.5	4.34	17.31	3.85	1.014	1.875
mean (*n* = 3)					− +71	±	0.2	0.06	0.36	0.09	0.051	0.014
h. Layer 4												
2018SCG05	4	Q13	91	95	+69		16.5	4.60	17.42	3.92	1.054	1.943
bulk sediment						±	0.3	0.02	0.30	0.11	0.093	0.027
i. Layer 6A												
2018SCG22	6A	Q17	61	27	+17		19.7	4.66	12.91	2.62	0.735	1.436
bulk sediment						±	0.1	0.02	0.23	0.07	0.064	0.017
2018SCG23	6A	Q17	60	27	+10		18.5	3.89	13.59	2.77	0.754	1.447
bulk sediment						±	0.1	0.02	0.25	0.07	0.066	0.018
bulk sediment	6A	Q17			+17		19.1	4.28	13.25	2.70	0.745	1.441
mean (*n* = 2)					− +10	±	0.6	0.39	0.34	0.07	0.046	0.012
j. Layer 6B1a												
2018SCG29	6B1a	P16	65	53	+5		17.7	9.89	18.28	2.82	1.015	2.218
bulk sediment						±	0.1	0.02	0.31	0.07	0.084	0.019
2018SCG25	6B1a	P16	60	55	+3		18.8	8.42	17.11	3.36	1.044	2.108
bulk sediment						±	0.1	0.02	0.31	0.09	0.088	0.022
2018SCG27	6B1a	Q15	90	20	−3		21.8	5.76	14.42	2.76	0.783	1.575
bulk sediment						±	0.1	0.02	0.26	0.07	0.067	0.017
2018SCG26	6B1a	P16	60	55	−5		19.0	8.85	15.32	2.84	0.945	1.968
bulk sediment						±	0.1	0.02	0.28	0.08	0.079	0.020
2018SCG30	6B1a	P16	65	52	−5		17.8	6.98	14.76	2.71	0.867	1.781
bulk sediment						±	0.1	0.02	0.26	0.07	0.073	0.017
bulk sediment	6B1a	P16			+5		19.0	7.98	15.98	2.90	0.931	1.930
mean (*n* = 4)		-Q15			− −5	±	1.5	1.45	1.48	0.24	0.096	0.230
k. Layer 6B1b												
2018SCG32	6B1b	P18	36	90	+9		19.4	3.68	17.43	2.91	0.786	1.594
bulk sediment						±	0.1	0.02	0.31	0.08	0.068	0.021
2018SCG34	6B1b	P18	98	89	+4		18.1	3.25	13.63	2.94	0.769	1.431
bulk sediment						±	0.1	0.02	0.26	0.08	0.068	0.020
2018SCG40	6B1b	P17	23	99	−1		17.9	3.51	15.73	3.05	0.814	1.568
bulk sediment						±	0.1	0.02	0.29	0.08	0.071	0.020
2018SCG33	6B1b	P18	37	89	−2		19.9	3.53	15.53	3.01	0.783	1.512
bulk sediment						±	0.1	0.02	0.27	0.09	0.070	0.020
bulk sediment	6B1b	P17			+9		18.9	3.51	15.64	2.98	0.789	1.530
mean (*n* = 4)		-P18			−2	±	0.3	0.12	0.91	0.04	0.068	0.040
l. Layer 6B2												
2018SCG41	6B2	Q16	2	30	−1		20.8	18.43	15.13	3.15	1.303	2.810
bulk sediment						±	0.1	0.02	0.28	0.08	0.104	0.019
2018SCG42	6B2	P17	23	99	−11		21.9	14.33	13.13	2.49	1.012	2.213
bulk sediment						±	0.1	0.02	0.23	0.07	0.082	0.016
2018SCG61	6B2	Q17	40	36	−20		21.2	29.73	13.56	2.14	1.481	3.531
bulk sediment						±	0.1	0.02	0.25	0.06	0.112	0.016
bulk sediment	6B2	Q16			−1		21.3	20.83	13.94	2.59	1.276	2.851
mean (*n* = 3)		-P17			−20	±	0.5	6.51	0.86	0.42	0.339	0.539
bulk sediment	6B2	Q16			−1		21.4	16.38	14.13	2.82	1.160	2.532
mean (*n* = 2)		-P17			−20	±	0.6	2.05	1.00	0.33	0.015	0.032
m. Layer 6B3a												
2018SCG45	6B3a	P15	60	30	−9		20.9	5.30	16.94	2.90	0.819	1.682
bulk sediment						±	0.1	0.02	0.30	0.08	0.070	0.019
2018SCG44a	6B3a	P15	47	25	−10		20.4	6.15	19.00	2.53	0.802	1.795
bulk sediment						±	0.1	0.02	0.33	0.07	0.068	0.019
2018SCG44b	6B3a	P15	47	25	−10		20.4	14.19	15.90	2.82	1.108	2.421
bulk sediment						±	0.1	0.02	0.30	0.07	0.089	0.018
bulk sediment	6B3a	P15			−9		20.6	8.55	17.28	2.75	0.910	1.966
mean (*n* = 3)					−10	±	0.2	4.01	1.29	0.16	0.140	0.325
bulk sediment	6B3a	P15			−9		20.7	5.79	17.99	2.71	0.810	1.742
mean (*n* = 2)					−10	±	0.2	0.43	1.03	0.19	0.010	0.014
n. Layer 6B3b												
2018SCG54	6B3b	Q16	45	97	−10		22.2	16.49	13.27	2.56	1.093	2.408
bulk sediment						±	0.1	0.02	0.24	0.07	0.088	0.016
2018SCG49	6B3b	Q17	40	30	−11		28.2	-	-	-	-	-
bulk sediment						±	0.2	-	-	-	-	-
2018SCG58	6B3b	Q17	70	45	−16		23.6	15.62	12.69	2.06	0.949	2.175
bulk sediment						±	0.1	0.02	0.23	0.05	0.075	0.013
2018SCG57	6B3b	Q17	4	15	−17		21.0	10.66	15.09	2.81	0.972	2.058
bulk sediment						±	0.1	0.02	0.26	0.07	0.080	0.017
2018SCG62	6B3b	Q17	17	65	−19		22.4	15.40	15.05	2.43	1.040	2.353
bulk sediment						±	0.1	0.02	0.26	0.06	0.083	0.016
2018SCG55	6B3b	Q16	80	79	−19		25.2	21.12	15.73	2.51	1.195	2.771
bulk sediment						±	0.1	0.02	0.28	0.07	0.094	0.016
bulk sediment	6B3b	Q16			−10		23.8	15.86	14.37	2.47	1.050	2.353
mean (*n* = 5 or 6)		-Q17			−19	±	2.4	3.33	1.17	0.24	0.089	0.244
bulk sediment	6B3b	Q16			−10		23.0	14.54	14.03	2.47	1.014	2.249
mean (*n* = 4)		-Q17			−19	±	0.9	2.28	1.07	0.27	0.070	0.140
o. Layer 6B3c												
2018SCG46	6B3c	P15	47	22	−19		20.8	5.76	17.24	2.91	0.839	1.739
bulk sediment						±	0.1	0.02	0.31	0.08	0.071	0.019
2018SCG59	6B3c	Q17	3	10	−26		21.1	7.74	14.68	2.44	0.801	1.715
bulk sediment						±	0.1	0.02	0.26	0.06	0.067	0.015
2018SCG63	6B3c	Q17	43	39	−30		21.7	9.19	16.17	2.50	0.862	1.896
bulk sediment						±	0.1	0.02	0.27	0.07	0.072	0.017
bulk sediment	6B3c	P15			−19		21.2	7.56	16.03	2.62	0.834	1.783
mean (*n* = 3)		-Q17			−30	±	0.4	1.41	1.05	0.21	0.025	0.080
p. Layer 7												
2018SCG04	7	Q13	75	95	+5		-	10.18	27.20	3.20	1.164	2.714
bulk sediment						±	-	0.02	0.44	0.08	0.097	0.027
2018SCG03	7	Q13	75	95	−6		17.1	8.00	22.90	2.78	0.976	2.249
bulk sediment						±	0.3	0.02	0.37	0.07	0.081	0.023
bulk sediment	7	Q13			+5		17.1	9.09	25.05	2.99	1.070	2.481
mean (*n* = 2)					−6	±	0.3	1.09	2.15	0.21	0.094	0.233
q. Grotto walls												
2018SCG01	-	debris	-	-	-		-	11.05	31.64	3.97	1.317	3.011
weathered roof		fall				±	-	0.02	0.51	0.11	0.147	0.209
2018SCG02	-	debris	-	-	-		-	5.81	25.34	5.60	1.621	2.963
unweathered roof		pile				±	-	0.02	0.42	0.13	0.142	0.033
roof rock	-		-	-	-		-	8.43	28.49	4.79	1.469	2.987
mean (*n* = 2)						±	-	2.62	3.15	0.82	0.152	0.024

^1^ Abbreviations: $D_{\mathrm{sed},\beta }^{\mathrm{BG}}\left(t\right)$ = the bulk sedimentary dose rate from *β* sources; $D_{\mathrm{sed},\gamma }^{\mathrm{BG}}\left(t\right)$ = the bulk sedimentary dose rate from γ sources; Concentrations and 1 σ errors were calculated for the closest tooth; ^2^ Calculated using the nearest tooth’s thicknesses and clastic sediment density, *ρ*_sed_ = 2.66 ± 0.01 g/cm^3^; enamel density, *ρ*_en_ = 2.95 ± 0.02 g/cm^3^; enamel density, *ρ*_d__en_ = 2.85 ± 0.02 g/cm^3^; ^3^ Calculated using cosmic dose rate, *D*_cos_(*t*) = 0.00 ± 0.00 μGy/y; ^4^ Values below detection limits: Assumed to be 0.00 ± 0.00 ppm for calculations.

**Table 3:** Dental Radioactivity at Saradj-Chuko Grotto, Russia.

Sample		U Concentrations (ppm)		
		Enamel	Inner Dentine	Outer Dentine
		[*U*_en_]	[*U*_inden_]	[*U*_outden_]
JT5, cheek tooth, Layer 6B1:				
JT5en1		- ^1^	136.78	136.12
JT5en2		- ^1^	149.66	151.08
JT5en3		- ^1^	162.38	158.35
JT5en4		- ^1^	156.85	161.71
Mean		- ^1^	151.42	151.82
	±	- ^1^	11.06	11.37
Typical concentration	~	0.01	0.01	0.01
uncertainties ^2^	-	0.02	0.02	0.02
Typical isotopic	~	0.01	0.01	0.01
detection limits ^2^	-	0.02	0.02	0.02
Typical water		0.02	0.05	0.05
concentrations (wt%) ^2^	±	0.02	0.02	0.02

^1^ Data not available. ^2^ Typical uncertainties, detection limits, and water concentrations depend on the tissue’s mass, tissue type, and diagenetic state.

## Appendix A

**Table A1:** ESR symbols and abbreviations.

Symbol	Definition
ESR	electron spin resonance (also called electron paramagnetic resonance, EPR)
HAP	hydroxyapatite, the major constituent mineral in bone, enamale, dentine, and dentinal cementum
𝒜_Σ_	the total accumulated radiation dose in the dated sample or subsample
*D*_Σ_(*t*)	the total dose rate from all sources for the dated sample or subsample
*D*_int_(*t*)	the dose rate from U, its daughters, and other radioisotopes inside the dated sample or subsample
*D*_ext_(*t*)	the dose rate from all sources outside the dated sample or subsample
*D*_sed_(*t*)	the dose rate from sedimentary U, Th, K, and other radioisotopes around the dated sample
*D*_sed,*β*_(*t*)	the dose rate from *β* particles from sedimentary U, Th, K, and other radioisotopes around the dated sample or subsample
*D*_sed,*γ*_(*t*)	the dose rate from γ radiation from sedimentary U, Th, K, and other radioisotopes around the dated sample or subsample
*D*_cos_(*t*)	the dose rate from cosmic sources affecting the dated sample or subsample
*t*_1_	the dated sample’s or subsample’s age
*t*_0_	today
*τ*	the mean ESR signal lifetime for an ESR signal
$\tau_{\mathrm{HAP}}^{}$	the mean ESR signal lifetime for the hydroxyapatite (HAP) signal in bone, enamel and other dentinal tissues
[*U*_en_]	the uranium concentration in the enamel for a dated tooth
[*U*_den_]	the uranium concentration in the dentine for a dated tooth
[*U*_inden_]	the uranium concentration in the inner dentine for a dated tooth
[*U*_outden_]	the uranium concentration in the outer dentine for a dated tooth
[*U*_sed_]	the uranium concentration in the sediment around a dated sample
[*U*_sed,*éb*_]	the uranium concentration in the *éboulis* (clasts generated by mass waste as the cave roof stopes upward) within the sediment around a dated sample
[*U*_sed,os_]	the uranium concentration in the other osseous components within the sediment around a dated sample, including in the bone, enamel, dentine, and/or dental cementum
[*U*_sed,igrx_]	the uranium concentration in the igneous rock clasts (e.g., *éboulis,* fluvial, or aeolian clasts) in the sediment around a dated sample, including any igneous rocks, tephra, volcaniclastic deposits, etc.
[*Th*_sed_]	the thorium concentration in the sediment around a dated sample
[*K*_sed_]	the potassium concentration in the sediment around a dated sample
[*W*_sed_(0)]	the modern water concentration measured now in the sediment around a dated sample
$\left[{\bar{W}}_{\mathrm{sed}}^{}\left(0\right)\right]$	the mean modern water concentration measured now in the sediment
[*W*_sed_(*t*)]	the water concentration in the sediment as a function of time around a dating sample
$\left[{\bar{W}}_{\mathrm{sed}}^{}\left(t\right)\right]$	the time-averaged water concentration in the sediment around a dating sample
*ρ*_en_	the density of the enamel in a dated tooth
*ρ*_den_	the density of the dentine in a dated tooth
*ρ*_sed_	the clastic sedimentary density around a dating sample
*p*	the U uptake rate (parameter) used in calculating *D*_int_(*t*)
EU	the early U uptake model used in calculating *D*_int_(*t*) with *p* = −1
LU	the linear (continuous) U uptake model used in calculating *D*_int_(*t*) with *p* = 0
RU	any recent U uptake model used in calculating *D*_int_(*t*), often generally used with *p* = 10
*D*_sed_(*t*_0_)	the modern sedimentary dose rate measured now for a dated sample
*D*_sed,*i*_(*t*)	the sedimentary dose rate derived from Layer *i*
*D*_sed,*j*_(*t*)	the sedimentary dose rate derived from Component *j*
*D*_sed,*i*,*j*_(*t*)	the sedimentary dose rate derived from Component *j* in Layer *i*
*D*_sed,*β*,*i*,*j*_(*t*)	the individual sedimentary dose rate derived from *β* sources within Component *j* in Layer *i*
*D*_sed,*γ*,*i*,*j*_(*t*)	the individual sedimentary dose rate derived from *γ* sources within Component *j* in Layer *i*
*D*_sed,*éb,β*_(*t*)	the sedimentary dose rate derived from *β* sources in the *éboulis* within the sediment
*D*_sed,*éb,γ*_(*t*)	the sedimentary dose rate derived from *γ* sources in the *éboulis* within the sediment
*D*_sed,os_(*t*)	the sedimentary dose rate derived from osseous components, including the bone, dentine, dental cementum
*D*_sed,clay_(*t*)	the sedimentary dose rate derived from the clay minerals
$D_{\mathrm{sed},\beta }^{\mathrm{BG}}\left(t\right)$	the sedimentary dose rate from *β* sources derived from bulk sedimentary geochemical analyses
$D_{\mathrm{sed},\gamma }^{\mathrm{BG}}\left(t\right)$	the sedimentary dose rate from γ sources derived from bulk sedimentary geochemical analyses
$D_{\mathrm{sed},\beta ,i}^{\mathrm{BG}}\left(t\right)$	the sedimentary dose rate from *β* sources derived from bulk sedimentary geochemical analyses in Layer *i*
$D_{\mathrm{sed},\gamma ,i}^{\mathrm{BG}}\left(t\right)$	the sedimentary dose rate from γ sources derived from bulk sedimentary geochemical analyses in Layer *i*
${\bar{D}}_{\mathrm{sed}}^{}\left(t\right)$	the time- and volumetrically averaged sedimentary dose rate for a dated sample
${\bar{D}}_{\mathrm{sed},\beta }^{}\left(t\right)$	the time- and volumetrically averaged sedimentary dose rate for a dated sample from *β* sources
${\bar{D}}_{\mathrm{sed},\beta ,i}^{}\left(t\right)$	the time- and volumetrically averaged sedimentary dose rate for a dated sample from *β* sources in Layer *i*
*D*_sed,*β*,*i*,*j*_(*t*)	the individual sedimentary dose rate derived from *β* sources within Component *j* in Layer *i*
*D*_sed,*γ*,*i*,*j*_(*t*)	the individual sedimentary dose rate derived from *γ* sources within Component *j* in Layer *i*
${\bar{D}}_{\mathrm{sed},\beta ,i,j}^{\mathrm{BG}}\left(t\right)$	the mean sedimentary dose rate derived from bulk analyses due to *β* sources within Component *j* in Layer *i*
${\bar{D}}_{\mathrm{sed},\gamma ,i,j}^{\mathrm{BG}}\left(t\right)$	the mean sedimentary dose rate derived from bulk analyses due to *γ* sources within Component *j* in Layer *i*
${\bar{D}}_{\mathrm{sed},\gamma }^{}\left(t\right)$	the time- and volumetrically averaged sedimentary dose rate for a dated sample from *γ* sources
${\bar{D}}_{\mathrm{sed},\gamma ,i}^{}\left(t\right)$	the time- and volumetrically averaged sedimentary dose rate for a dated sample from *γ* sources in Layer *i*
${\bar{D}}_{\cos}^{}\left(t\right)$	the time- and volumetrically averaged sedimentary dose rate for a dated sample
𝒜_sat_	the accumulated radiation dose in the dated sample or subsample at signal saturation
LD_50_	the lethal dose for 50% of humans tested
X	the north-south position within a square for a tooth or sediment sample relative to the (0,0,0) cave datum
Y	the east-west position within a square for a tooth or sediment sample relative to the (0,0,0) cave datum
Z	the depth for a tooth or sediment sample relative to the (0,0,0) cave datum
MIS	Marine (Oyygen) Isotope Stage

## References

[1] Rink W.J. (2001). Beyond ^14^C dating: A user’s guide to long-range dating methods in archaeology. Earth Sci. Archaeol..

[2] Skinner A.R., Rink W.J., Thompson J.W. (2015). General principles of electron spin resonance (ESR) dating. The Encyclopedia of Scientific Dating Methods.

[3] Skinner A.R., Blackwell B.A.B., Chasteen D.E., Shao J.M., Min S.S. (2000). Improvements in dating tooth enamel by ESR. Appl. Radiat. Isot..

[4] Skinner A.R., Blackwell B.A.B., Chasteen D.E., Shao J.M. (2001). Q-band ESR studies of fossil tooth enamel. Quat. Sci. Rev. (Quat. Geochronol.).

[5] Blackwell B.A.B. (2006). Electron spin resonance (ESR) dating in karst environments. Acta cars..

[6] Blackwell B.A.B., Skinner A.R., Blickstein J.I.B., Montoya A.C., Florentin J.A., Baboumian S.M., Ahmed I.J., Deely A.E. (2016). ESR in the 21st Century: From buried valleys and deserts to the deep ocean and tectonic uplift. Earth Sci. Rev..

[7] Deely A.E., Blackwell B.A.B., Mylroie J.E., Carew J.L., Blickstein J.I.B., Skinner A.R. (2011). Testing cosmic dose rate models for ESR: Dating corals and molluscs on San Salvador, Bahamas. Radiat. Meas..

[8] Blackwell B.A.B., Šalamanov-Korobar L., Huang C.L.C., Zhuo J.L., Kitanovski B., Blickstein J.I.B., Florentin J.A., Vasilevski S. (2019). Hunting elusive sedimentary U and Th in an Upper Paleolithic-Middle Paleolithic (MP-UP) transition site: Increasing ESR tooth dating accuracy at Golema Pešt, Macedonia. Radiat. Prot. Dosim..

[9] Greeley R. (1987). The role of lava tubes in Hawai’ian volcanoes. US Geol. Surv. Prof. Pap..

[10] Kennedy J., Brady J.E. (1997). Into the nether world of Island Earth: A reevaluation of refuge caves in ancient Hawai’ian society. Geoarchaeol..

[11] Lundburg J., McFarlane D.A., Harmon R.S., Wicks C. (2006). Speleogenesis of the Mount Elgon elephant caves, Kenya. Perspectives on Karst Geomorphology, Hydrology, and Geochemistry: A Tribute to Derek C. Ford and William B. White.

[12] Forti P., Galli E., Rossi A. (1998). Minerogenesis of volcanic caves of Kenya. Int. J. Speleol..

[13] Willoughby P.R., Compton T., Bello S.M., Bushozi P.M., Skinner A.R., Stringer C.B. (2018). Middle Stone Age human teeth from Magubike Rock Shelter, Iringa Region, Tanzania. PLoS One.

[14] Crawford R.L. (1996). The world’s longest lava tube caves: Third revision. J. Speleol. Soc. Korea.

[15] Brennan B.J., Schwarcz H.P., Rink W.J. (1997). Simulation of the γ radiation field in lumpy environments. Radiat. Meas..

[16] Blackwell B.A.B., Blickstein J.I.B. (2000). Considering sedimentary U uptake in external dose rate determinations for ESR and luminescent dating. Quat. Int..

[17] Doronicheva E.V., Golovanova L.V., Doronichev V.B., Nedomolkin A.G., Shackley M.S. (2017). The first Middle Paleolithic site exhibiting obsidian industry on the northern slopes of the central Caucasus. Antiquity.

[18] Doronicheva E.V., Golovanova L.V., Doronichev V.B., Nedomolkin A.G., Korzinova A.S., Tselmovitch V.A., Kulkova M.A., Odinokova E.V., Shirobokov I.G., Ivanov V.V. (2019). The first laminar Mousterian obsidian industry in the north-central Caucasus, Russia: Preliminary results of multi-disciplinary research at Saradj-Chuko Grotto. Archaeol. Res. Asia.

[19] Doronicheva E.V., Golovanova L.V., Doronichev V.B., Shackley M.S., Nedomolkin A.G. (2019). New data about exploitation of the Zayukovo (Baksan) obsidian source in northern Caucasus during the Paleolithic. J. Archaeol. Sci..

[20] Kizevalter D.S., Karpinsky A.P. (1959). SheetK-38-II. The Geological Map of the USSR 1959, Scale 1:200,000.

[21] Blackwell B.A. (1989). Laboratory Procedures for ESR Dating of Tooth Enamel. McMaster Univ. Dept. Geol. Tech. Memo.

[22] Adamiec G., Aitken M.J. (1998). Dose rate conversion factors: Update. Anc. TL.

[23] Brennan B.J., Rink W.J., McGuirl E.L., Schwarcz H.P. (1997). β doses in tooth enamel by“one-group”theory and the Rosy ESR dating software. Radiat. Meas..

[24] Grün R. (2009). The DATA program for the calculation of ESR age estimates on tooth enamel. Quat. Geochronol..

[25] Dibble H.L., Aldaeias V., Alvarez-Fernàndez E., Blackwell B.A.B., Hallett-Desguez E., Jacobs Z., Goldberg P., Lin S.C., Morala A., Meyer M.C. (2012). New excavations at the site of Contrebandiers Cave, Morocco. Paleoanthrop..

[26] Blackwell B.A.B., Skinner A.R., Brassard P., Blickstein J.I.B., Whitehead N.E., Ikeya M. (2002). U uptake in tooth enamel: Lessons from isochron analyses and laboratory simulation experiments. Proceedings of the International Symposiumon New Prospects in ESR Dosimetry and Dating.

[27] Blackwell B.A.B., Liang S.S., Golovanova L.V., Doronichev V.B., Skinner A.R., Blickstein J.I.B. (2005). ESR at Treugol’naya Cave, northern Caucasus Mt., Russia: Dating Russia’s oldest archaeological site and paleoclimatic change in Oxygen Isotope Stage 11. Appl. Radiat. Isot..

[28] Blackwell B.A.B., Yu E.S.K., Skinner A.R., Turk I., Blickstein J.I.B., Turk J., Yin V.S.W., Lau B., Turk I. (2007). ESR-datiranje najdišč a Divje babe I, Slovenija. Divje babe I: Paleotiscko Najdišče Mlajšega Pleistocena v Sloveniji (Divje babe I: Upper Pleistocene Palaeolithic Site in Slovenia), Vol. 1: Geologija in paleontologija (Geology and Paleontology).

[29] Blackwell B.A.B., Yu E.S.K., Skinner A.R., Turk I., Blickstein J.I.B., Turk J., Yin V.S.W., Lau B., Turk I. (2007). ESR dating at Divje Babe I, Slovenija. Divje babe I: Paleotiscko Najdišče Mlajšega Pleistocena v Sloveniji (Divje babe I: Upper Pleistocene Palaeolithic Site in Slovenia), Vol. 1: Geologija in Paleontologija (Geology and Paleontology).

[30] Blackwell B.A.B., Skinner A.R., Blickstein J.I.B., Golovanova L.V., Doronichev V.B., Séronie-Vivien M.R., Camps M., Chauhan P.R. (2009). ESR dating at hominid and archaeological sites during the Pleistocene. The Sourcebook for Paleolithic Transitions.

[31] Blackwell B.A.B., Huang Y.E.W., Chu S.M., Mihailović D., Roksandic M., Dimitrijević V., Blickstein J.I.B., Skinner A.R., Mihailović D. (2014). ESR dating tooth enamel from the Mousterian Layers at Pešturina Cave, Serbia. Paleolithic and Mesolithic Research in the Central Balkans.

[32] Skinner A.R., Blackwell B.A.B., Mian A., Baboumian S., Blickstein J.I.B., Wrinn P.J., Krivoshapkin A.I., Derevianko A.P., Lundburg J.A. (2007). ESR analyses on tooth enamel from the Paleolithic layers at the Obi-Rakhmat hominid site, Uzbekistan: Tackling a dating controversy. Radiat. Meas..

[33] Skinner A.R., Blackwell B.A.B., Martin S.A., Ortega A.J., Blickstein J.I.B., Golovanova L.V., Doronichev V.B. (2005). ESR dating at Mezmaiskaya Cave, Russia. Appl. Radiat. Isot..

[34] Cherdyntsev V.V., Schmorak J. (1971). Uranium-234 (Uran-234), Israel Program for Scientific Translations.

[35] Gascoyne M., Ivanovich M., Harmon R.S. (1992). Geochemistry of the actinides and their daughters. Uranium Series Disequilibrium: Application to Environmental Problems.

[36] Ivanovich M., Harmon R.S. (2007). Uranium Series Disequilibrium: Application to Environmental Problems.

[37] Osmond J.K., Ivanovich M., Ivanovich M., Harmon R.S. (1992). Uranium-series mobilization and surface hydrology. Uranium Series Disequilibrium: Application to Environmental Problems.

[38] Brassard P., Skinner A.R., Blackwell B.A.B., Blickstein J.I.B. (2002). Specific sorption of uranium in modern and fossil dentine. International Conference on Luminescent & ESR Dating (LED).

[39] Grün R., Taylor L. (1996). Uranium and thorium in the constituents of fossil teeth. Anc.TL.

[40] ICRP (2007). The Recommendations of the International Commissionon Radiological Protection. IRCP103. Ann. ICRP.

